# Upregulation of LIMK1 Is Correlated With Poor Prognosis and Immune Infiltrates in Lung Adenocarcinoma

**DOI:** 10.3389/fgene.2021.671585

**Published:** 2021-06-03

**Authors:** Guojun Lu, Ying Zhou, Chenxi Zhang, Yu Zhang

**Affiliations:** ^1^Department of Respiratory Medicine, Nanjing Chest Hospital, Affiliated Nanjing Brain Hospital, Nanjing Medical University, Nanjing, China; ^2^Central Laboratory, Nanjing Chest Hospital, Affiliated Nanjing Brain Hospital, Nanjing Medical University, Nanjing, China

**Keywords:** lung adenocarcinoma, *LIMK1*, LIM domain kinase1, biomarker, prognosis, immune infiltrates

## Abstract

**Background:**

Protein-coding gene LIM Domain Kinase 1 (*LIMK1*) is upregulated in various tumors and reported to promote tumor invasion and metastasis. However, the prognostic values of *LIMK1* and correlation with immune infiltrates in lung adenocarcinoma are still not understood. Therefore, we evaluated the prognostic role of *LIMK1* and its correlation with immune infiltrates in lung adenocarcinoma.

**Methods:**

Transcriptional expression profiles of *LIMK1* between lung adenocarcinoma tissues and normal tissues were downloaded from the Cancer Genome Atlas (TCGA). The LIMK1 protein expression was assessed by the Clinical Proteomic Tumor Analysis Consortium (CPTAC) and the Human Protein Atlas. Receiver operating characteristic (ROC) curve was used to differentiate lung adenocarcinoma from adjacent normal tissues. Kaplan-Meier method was conducted to assess the effect of *LIMK1* on survival. Protein-protein interaction (PPI) networks were constructed by the STRING. Functional enrichment analyses were performed using the “ClusterProfiler” package. The relationship between *LIMK1* mRNA expression and immune infiltrates was determined by tumor immune estimation resource (TIMER) and tumor-immune system interaction database (TISIDB).

**Results:**

The expression of *LIMK1* in lung adenocarcinoma tissues was significantly upregulated than those in adjacent normal tissues. Increased *LIMK1* mRNA expression was associated with lymph node metastases and high TNM stage. The ROC curve analysis showed that with a cutoff level of 4.908, the accuracy, sensitivity, and specificity for *LIMK1* differentiate lung adenocarcinoma from adjacent controls were 69.5, 93.2, and 71.9%, respectively. Kaplan-Meier survival analysis showed lung adenocarcinoma patients with high- *LIMK1* had a worse prognosis than those with low- *LIMK1* (43.1 vs. 55.1 months, *P* = 0.028). Correlation analysis indicated *LIMK1* mRNA expression was correlated with tumor purity and immune infiltrates.

**Conclusion:**

Upregulated *LIMK1* is significantly correlated with poor survival and immune infiltrates in lung adenocarcinoma. Our study suggests that *LIMK1* can be used as a biomarker of poor prognosis and potential immune therapy target in lung adenocarcinoma.

## Introduction

Lung cancer is one of the most common malignant tumors around the world and the leading cause for cancer-related death (Jemal et al., 2011). The incidence of lung cancer has steadily increased over recent years. Lung cancer remains refractory and the 5-year survival rate continues to be the lowest among the major cancers. It is speculated that numerous people will be diagnosed with lung cancer in the future, which bring a heavy economic burden to our society (Torre et al., 2016; Albaba et al., 2017). In the subtypes of lung cancer, lung adenocarcinoma accounts for about 50% (Brustugun et al., 2018). Despite many therapeutic endeavors has been made in lung adenocarcinoma, such as targeted therapy and immunotherapy, the survival rate remains bleak and staggers at about 20% 5 years after treatment (Hirsch et al., 2017). Thus, it is imperative to search novel biomarkers for advancing the prognosis of lung adenocarcinoma.

LIM Domain Kinase 1 (*LIMK1*) is a protein known as a member of the LIM kinase protein family. *LIMK1* is consisted of gene spans 39,499 base pairs with 16 exons and encoded by a gene located on human chromosome 7q11.23 (Scott and Olson, 2007). Through phosphorylation and inactivation to its downstream effector of cofilin, *LIMK1* has been shown to be important in regulating the polymerization of actin (Liu et al., 2019). When *LIMK1* is phosphorylated, cofilin loses the ability to bind to actin, leading to the accumulation of actin polymers dysregulation of actin-mediated cytoskeletal changes (Nishimura et al., 2006). The phosphorylation of *LIMK1* has been implicated with many cellular functions including angiogenesis, proliferation, cell cycle, and metastasis progression (Foletta et al., 2004; Nishimura et al., 2006). Previous studies have confirmed that ectopic expression of *LIMK1* was associated with the progression of several tumor types, such as colorectal cancer, gastric cancer, prostate cancer, and breast cancer (Davila et al., 2003; McConnell et al., 2011; You et al., 2015; Liao et al., 2017). A paper from Huang et al. (2020) indicated that the upregulation of *LIMK1* is correlated with lymph node metastasis and poor biochemical-free survival in prostate cancer. In pancreatic cancer, Vlecken and Bagowski (2009) reported that knockdown of *LIMK1* can lead to an inhibition of invasion and metastatic behavior, as well as suppression of pancreatic cancer cell-induced angiogenesis. Moreover, some recent findings suggested that downregulation of *LIMK1* can inhibit lung cancer cell migration (Chen et al., 2013; Wan et al., 2014; Zhang et al., 2020). Thus, *LIMK1* has great potential to be a biomarker of poor prognosis and therapeutic target for lung cancer.

The prognostic values and correlation with immune infiltrates of *LIMK1* in lung adenocarcinoma are still not fully understood. Given the overexpression of *LIMK1* in lung cancer and the downregulation of *LIMK1* can inhibit lung cancer cell migration, we hypothesized that the level of *LIMK1* is associated with survival in lung adenocarcinoma. To test this hypothesis, we evaluated the prognostic role of *LIMK1* in lung adenocarcinoma based on data from The Cancer Genome Atlas (TCGA). In this study, we found that *LIMK1* is upregulated in lung adenocarcinoma. Significantly, the upregulation of *LIMK1* is correlated with poor clinical characteristics and risk factors. We further evaluated the diagnostic and prognostic values, the correlation with immune infiltrates of *LIMK1* for lung adenocarcinoma. Our study links the overexpression of *LIMK1* and poor survival in lung adenocarcinoma.

## Materials and Methods

### TCGA Datasets

Transcriptional expression data of *LIMK1* and corresponding clinical information were downloaded from TCGA official website^1^ (Tomczak et al., 2015). The 18 enrolled cancer types contained at least 5 samples in the normal group. Finally, the RNA-Seq gene expression data with workflow type of FPKM was transformed into TPM format and log2 conversion for further study. Since all the data were downloaded from TCGA, this study did not need approval from the Ethics Committee.

### RNA-Sequencing Data of *LIMK1* in Lung Adenocarcinoma

The RNA-Seq expression data of *LIMK1* in lung adenocarcinoma was also downloaded from TCGA. Therefore, 535 lung adenocarcinoma and 59 adjacent normal tissue data were retained. The samples selected contained *LIMK1* gene expression data and associated clinical information, including age, gender, smoker condition, T stage, N stage, M stage, and tumor location. The mRNA expression data were characterized by mean ± *SD*.

### Clinical Proteomic Tumor Analysis Consortium (CPTAC) and UALCAN

With the application of proteomic technologies, CPTAC^2^ analyzes tumor biospecimens using mass spectrometry, quantifying and identifying the constituent proteins and characterizing proteome of each tumor sample (Edwards et al., 2015). UALCAN^3^ is a user-friendly online web resource for analyzing publicly available cancer data (Chandrashekar et al., 2017). In this study, we performed UALCAN to present a throughout analysis of LIMK1 protein expression from CPTAC.

### The Human Protein Atlas (HPA)

HPA^4^ contains normal tissues and tumor tissues information regarding the expression profiles of human genes on protein level (Uhlen et al., 2015, 2017). In this study, we conducted HPA to compare the protein expression of LIMK1 between normal lung tissue and lung adenocarcinoma tissue.

### Protein-Protein Interaction (PPI) Networks and Functional Enrichment Analysis

STRING is an online database for the retrieval of interacting genes (version 11.0^5^; Szklarczyk et al., 2011). In this study, we conducted STRING to search co-expression genes and construct PPI networks with an interaction score >0.4. Gene ontology (GO) enrichment and Kyoto Encyclopedia of Genes and Genomes (KEGG) pathway analyses of co-expression genes were performed by the “ClusterProfiler” package and visualized by the “ggplot2” package (Wickham, 2016; Yu et al., 2012).

### Tumor Immune Estimation Resource (TIMER) Database

TIMER is a comprehensive online resource for systematic analysis of immune infiltrates across various cancer types^6^ (Li et al., 2017). In this study, we performed TIMER to determine the relationship between *LIMK1* expression in lung adenocarcinoma and six immune infiltrates (B cells, CD4^+^ T cells, CD8^+^ T cells, neutrophils, macrophages, and dendritic cells).

### Tumor-Immune System Interaction Database (TISIDB)

TISIDB^7^ is an online web integrated repository portal for tumor-immune system interaction (Ru et al., 2019). In this study, we performed TISIDB to determine the expression of *LIMK1* and tumor-infiltrating lymphocytes (TILs) across human cancers. Based on the gene expression profile, the relative abundance of TILs was inferred by using gene set variation analysis. The correlations between *LIMK1* and TILs were measured by Spearman’s test.

### PrognoScan Database

PrognoScan database^8^ is a powerful online platform to evaluate the correlation between gene expression and survival across various types of cancers (Mizuno et al., 2009). In this study, we performed PrognoScan database to analyze the correlation between *LIMK1* expression and overall survival in lung adenocarcinoma with two different datasets (jacob-00182-CANDF, jacob-00182-MSK).

### Statistical Analyses

All statistical analyses were performed with R (V 3.6.3)^9^ and R package ggplot2 was used to visualize expression differences. Paired *t*-test and Mann-Whitney *U*-test were used to determine the differences between lung adenocarcinoma tissues and adjacent normal tissues. ROC curve was performed to detect the cutoff value of *LIMK1* using the pROC package (Robin et al., 2011). Kaplan-Meier and log-rank tests were conducted with the survminer package^10^ to assess the effect of *LIMK1* on survival.

## Results

### Expression Pattern of *LIMK1* in Pan-Cancer Perspective

**FIGURE 1:**
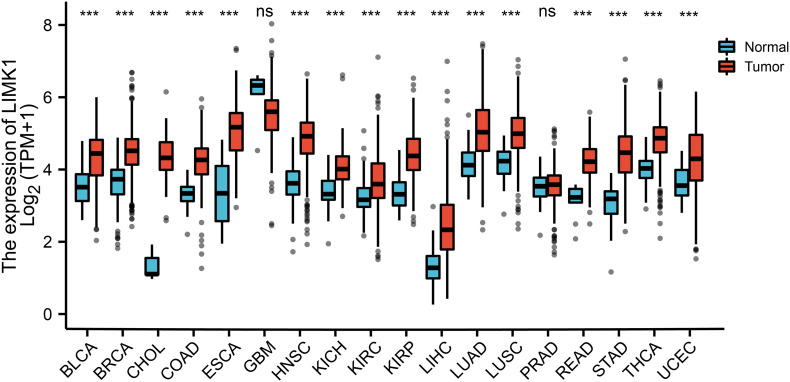
Expression pattern of *LIMK1* in Pan-cancer perspective. The mRNA expression of *LIMK1* was upregulated in 16 of 18 cancer types compared with normal tissues. (***P < 0.001). ns, no significance; BLCA, bladder urothelial carcinoma; BRCA, breast invasive carcinoma; CHOL, cholangiocarcinoma; COAD, colon adenocarcinoma; ESCA, esophageal carcinoma; GBM, glioblastoma mutiforme; HNSC, head and neck squamous cell carcinoma; KICH, kidney chromophobe; KIRC, kidney renal clear cell carcinoma; KIRP, kidney renal papillary cell carcinoma; LIHC, liver hepatocellular carcinoma; LUAD, lung adenocarcinoma; LUSC, lung squamous cell carcinoma; PRAD, prostate adenocarcinoma; READ, rectum adenocarcinoma; STAD, stomach adenocarcinoma; THCA, thyroid carcinoma; UCEC, uterine corpus endometrial carcinoma.

To evaluate the mRNA expression pattern of *LIMK1* across different cancer types, we excluded from the analysis the datasets from 15 cancer types that contained less than five samples in the normal group. The final working set refers to 18 cancer types. As shown in Figure 1, compared with normal tissues, *LIMK1* was significantly upregulated in 16 of all 18 cancer types. This data indicated the mRNA expression of *LIMK1* was abnormally expressed across different cancer types.

### Upregulated mRNA and Protein Expression of LIMK1 in Patients With Lung Adenocarcinoma

**FIGURE 2:**
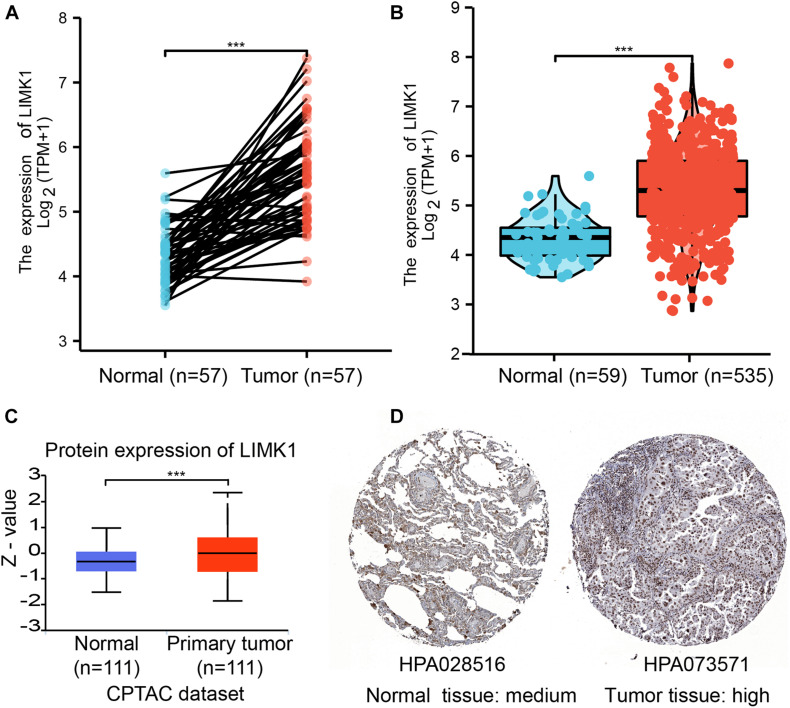
The mRNA and protein expression of LIMK1 in lung adenocarcinoma. **(A)** The mRNA expression levels of *LIMK1* in 57 lung adenocarcinoma and matched-adjacent normal samples. **(B)** The mRNA expression levels of *LIMK1* in 535 lung adenocarcinoma samples and 59 normal samples. **(C)** The protein expression levels of LIMK1 based on CPTAC. **(D)** The protein levels of LIMK1 based on Human Protein Atlas. Normal tissue, https://www.proteinatlas.org/ENSG00000106683-LIMK1/tissue/lung#img; Tumor tissue, https://www.proteinatlas.org/ENSG00000106683-LIMK1/pathology/lung+cancer#img (***P < 0.001).

To determine the mRNA and protein expression of LIMK1 in lung adenocarcinoma, the LIMK1 expression data from TCGA and HPA were analyzed. The baseline characteristics of lung adenocarcinoma patients from TCGA were listed in Supplementary Table 1. As shown in Figure 2A, paired data analysis showed that the mRNA expression levels of *LIMK1* in lung adenocarcinoma tissues (*n* = 57) were significantly higher than those in adjacent normal tissues (*n* = 57) (Figure 2A, 5.584 ± 0.747 vs. 4.320 ± 0.442, *P* < 0.001). Unpaired data analyses also showed that the mRNA expression levels of *LIMK1* in lung adenocarcinoma tissues (*n* = 535) were significantly higher than those in adjacent normal tissues (*n* = 59) (Figure 2B, 5.314 ± 0.847 vs. 4.324 ± 0.437, Mann-Whitney *U*-test, *P* < 0.001). To present a throughout analysis of LIMK1 protein expression, we performed analysis on CPTAC with UALCAN. The result showed that the protein expression of LIMK1 in lung adenocarcinoma was significantly higher than those in normal tissues (Figure 2C). As shown in Figure 2D, immunohistochemical staining from HPA also revealed LIMK1 protein was upregulated in lung adenocarcinoma tissue. These results indicated that both mRNA and protein expression of LIMK1 are upregulated in lung adenocarcinoma tissues.

### Relationships Between *LIMK1* mRNA Levels and Clinical Pathological Characteristics of Lung Adenocarcinoma Patients

**FIGURE 3:**
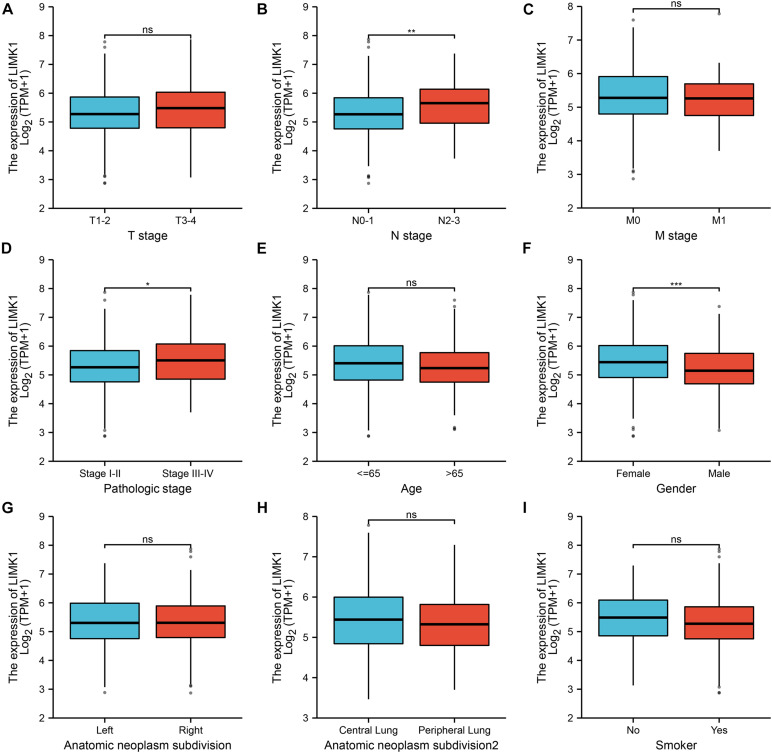
Relationships between *LIMK1* mRNA levels and clinical pathological characteristics. *LIMK1* mRNA expression was significantly correlated with lymph node metastases **(B)**, high TNM stage **(D)** and gender was male **(F)**. However, no statistically significant correlation were found between the expression levels of *LIMK1* and T stage **(A)**, M stage **(C)**, age **(E)**, anatomic neoplasm subdivision **(G,H)** and smoke condition **(I)** (ns, no significance, *P < 0.05, **P < 0.01, ***P < 0.001).

To evaluate the association between the mRNA expression of *LIMK1* and clinical pathological characteristics of lung adenocarcinoma samples, we performed Mann-Whitney *U*-test and logistic regression analysis. As shown in Table 1 and Figures 3A–I, higher expression levels of *LIMK1* were observed in male patients (*P* = 0.004), patients with lymph node metastases (*P* = 0.022), and patients with high TNM stage (*P* = 0.048). However, no statistically significant correlation were found between the expression levels of *LIMK1* and other clinical pathological characteristics, such as age (*P* = 0.113), smoker (*P* = 0.270), T stage (*P* = 0.129), M stage (*P* = 0.921), and anatomic subdivision (right vs. left, *P* = 0.959; peripheral vs. central, *P* = 0.562). Taken together, these results suggested that *LIMK1* is correlated with lymph node metastases and high TNM stage, further suggesting *LIMK1* may act as a biomarker of poor prognosis for lung adenocarcinoma.

**TABLE 1:** Clinical characteristics of the lung adenocarcinoma patients (TCGA).

Characteristics	Total	Low expression	High expression	*P-*value
		
	*N* (%)	*N* (%)	*N* (%)	
**T stage**				0.199
T1	175 (32.9%)	97 (18.2%)	78 (14.7%)	
T2	289 (54.3%)	140 (26.3%)	149 (28%)	
T3	49 (9.2%)	21 (3.9%)	28 (5.3%)	
T4	19 (3.6%)	7 (1.3%)	12 (2.3%)	
**N stage**				0.006**
N0	348 (67.0%)	188 (36.2%)	160 (30.8%)	
N1	95 (18.3%)	39 (7.5%)	56 (10.8%)	
N2	74 (14.3%)	28 (5.4%)	46 (8.9%)	
N3	2 (0.4%)	0 (0%)	2 (0.4%)	
**M stage**				1.000
M0	361 (93.5%)	184 (47.7%)	177 (45.9%)	
M1	25 (6.5%)	13 (3.4%)	12 (3.1%)	
**Pathologic stage**				0.002**
Stage I	294 (55.8%)	165 (31.3%)	129 (24.5%)	
Stage II	123 (23.3%)	50 (9.5%)	73 (13.9%)	
Stage III	84 (16.0%)	31 (5.9%)	53 (10.1%)	
Stage IV	26 (4.9%)	14 (2.7%)	12 (2.3%)	
**Gender**				0.005**
Female	286 (53.5)	126 (23.6%)	160 (29.9%)	
Male	249 (46.5)	141 (26.4%)	108 (20.2%)	
**Age**				0.134
< = 65	255(49.4)	118 (22.9%)	137 (26.6%)	
>65	261(50.6)	139 (26.9%)	122 (23.6%)	
**Smoker**				0.327
No	75(14.4)	33 (6.3%)	42 (8.1%)	
Yes	446(85.6)	227 (43.6%)	219 (42%)	
**Anatomic neoplasm subdivision**				1.000
Left	205(39.4)	102 (19.6%)	103 (19.8%)	
Right	315 (60.6)	156 (30%)	159 (30.6%)	
**Anatomic neoplasm subdivision 2**				0.671
Central lung	62 (32.8)	27 (14.3%)	35 (18.5%)	
Peripheral lung	127 (67.2)	61 (32.3%)	66 (34.9%)	

**P < 0.01.

### Differential RNA-Seq Levels of *LIMK1* as a Prospective Biomarker to Distinguish Lung Adenocarcinoma Samples From Normal Samples

**FIGURE 4:**
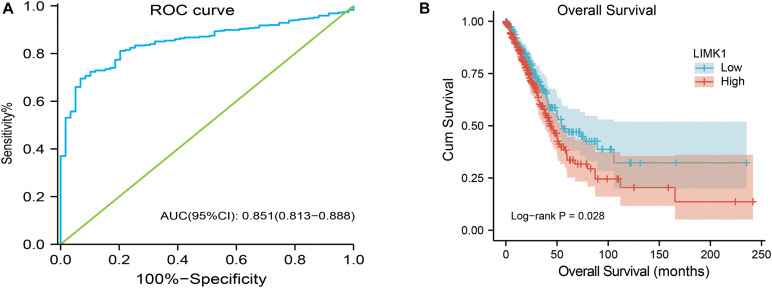
ROC and Kaplan-Meier curves for *LIMK1*. **(A)** ROC curve showed that *LIMK1* had an AUC value of 0.851 to discriminate lung adenocarcinoma tissues from healthy controls. With a cutoff of 4.908, the sensitivity, specificity and accuracy were 93.2, 71.9, and 69.5%, respectively. **(B)** Kaplan-Meier survival curves indicated that lung adenocarcinoma patients with high *LIMK1* mRNA expression had a shorter OS than those with low-level of *LIMK1* (43.1 vs. 55.1 months, *P* = 0.028).

To investigate the value for *LIMK1* to distinguish lung adenocarcinoma samples from normal smples, we performed a ROC curve analysis. As showed in Figure 4A, the ROC curve analysis showed *LIMK1* had an AUC value of 0.851 (95% CI: 0.813–0.888). At a cutoff of 4.908, *LIMK1* had a sensitivity, specificity, and accuracy of 69.5, 93.2, and 71.9%, respectively. The positive predictive value was 98.9% and the negative predictive value was 25.2%. These findings indicated that *LIMK1* could be a promising biomarker to differentiate lung adenocarcinoma tissues from normal tissues.

### High mRNA Expression of *LIMK1* Is Associated With Short OS

To explore the relationship between *LIMK1* mRNA expression and OS in lung adenocarcinoma patients, Kaplan-Meier curves and PrognoScan database were performed. As shown in Figure 4B, the OS of lung adenocarcinoma patients with high-level of *LIMK1* was significantly shorter than those with low-level of *LIMK1* (43.1 vs. 55.1 months, *P* = 0.028). PrognoScan result with two different datasets (Supplementary Figure 1) also indicated that high expression of *LIMK1* was correlated with poor overall survival in lung adenocarcinoma. These data indicated that high mRNA expression of *LIMK1* is a biomarker of poor prognosis in lung adenocarcinoma.

### PPI Networks and Functional Annotations

**FIGURE 5:**
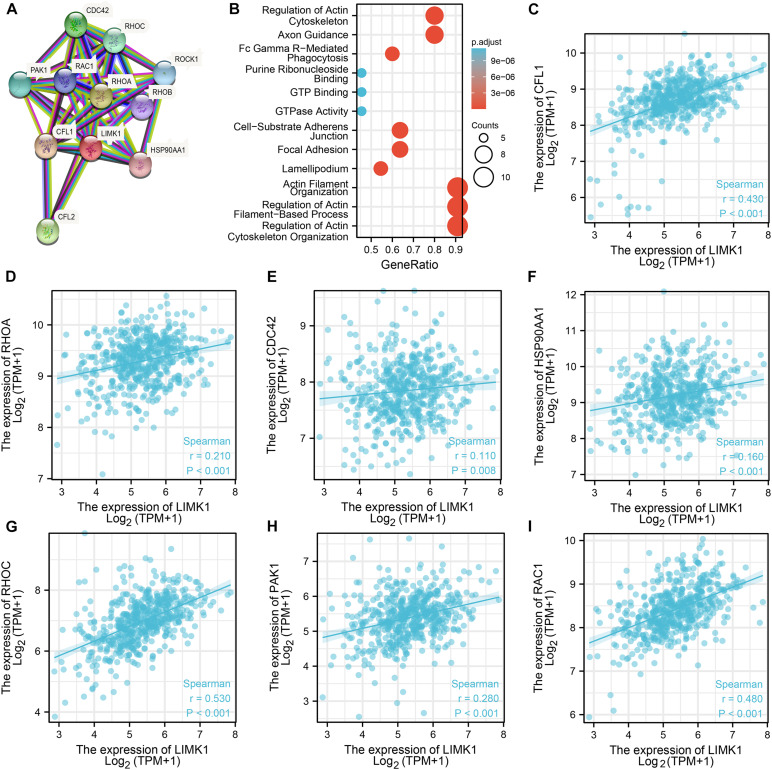
PPI networks and functional enrichment analyses. **(A**) A network of *LIMK1* and its co-expression genes. **(B)** Functional enrichment analyses of 11 involved genes. *LIMK1* was associated with actin filament organization, regulation of actin filament-based process, and actin cytoskeleton organization. These genes were involved in purine ribonucleoside binding, GTP Binding, and GTPase Activity. **(C–I)** The correlation analyses between the expression of *LIMK1* and co-expressed genes in lung adenocarcinoma.CFL1, cofilin-1; RHOA, transforming protein RhoA; CFL2, cofilin-2; CDC42, cell division control protein 42 homolog; RHOC, Rho-related GTP-binding protein RhoC; PAK1, serine/threonine-protein kinase PAK 1; ROCK1, Rho-associated protein kinase 1; RAC1, Ras-related C3 botulinum toxin substrate 1; RHOB, Rho-related GTP-binding protein RhoB; HSP90AA1, heat shock protein HSP 90-alpha.

To construct PPI networks and functional annotations, we conducted STRING database, GO, and KEGG analyses. Figure 5A showed a network of *LIMK1* and its 10 co-expression genes. As shown in Figure 5B, changes in the biological process of *LIMK1* were associated with actin filament organization, regulation of actin filament-based process, and actin cytoskeleton organization. Functional annotations indicated that these genes were involved in purine ribonucleoside binding, GTP Binding, and GTPase activity. The correlation analyses between the expression of *LIMK1* and co-expressed genes in lung adenocarcinoma from TCGA were shown in Figures 5C–I.

### Correlation Analysis Between *LIMK1* Expression and Immune Cell Infiltration in Lung Adenocarcinoma

**FIGURE 6:**
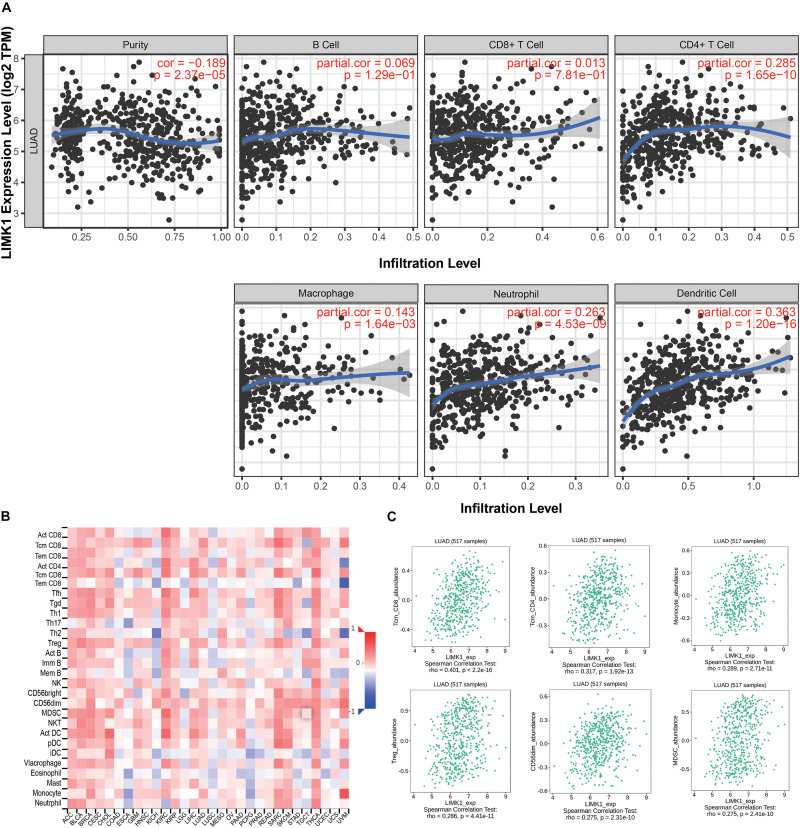
Correlations of *LIMK1* expression with immune infiltration level. **(A)** *LIMK1* expression is negatively related to tumor purity and has correlations with dendritic cell, CD4^+^ T cell, neutrophil, and macrophage in lung adenocarcinoma. **(B)** Relations between the expression of *LIMK1* and 28 types of TILs across human cancers. **(C)** *LIMK1* was correlated with abundance of CD8^+^ T cells, CD4^+^ T cells, monocyte cells, Treg cells, CD56dim cells, and MDSC cells.

We analyzed the correlation between *LIMK1* expression and the six types of tumor infiltrating immune cells in the TIMER database. As shown in Figure 6A, *LIMK1* expression had correlations with tumor purity (*r* = −0.189, *P* = 2.37e-05), CD4^+^ T cell (*r* = 0.285, *P* = 1.65e-10), macrophage (*r* = 0.143, *P* = 1.64e-03), neutrophil (*r* = 0.263, *P* = 4.53e-09), dendritic cell (*r* = 0.363, *P* = 1.20e-16). We also evaluated the correlation between *LIMK1* expression and 28 types of TILs in the TISIDB database. Figure 6B shown the relations between expression of *LIMK1* and 28 types of TILs across human cancers. As shown in Figure 6C, the expression of *LIMK1* was correlated with abundance of CD8^+^ T cells (*r* = 0.401, *P* = 2.2e-16), CD4^+^ T cells (*r* = 0.317, *P* = 1.92e-16), monocyte cells (*r* = 0.289, *P* = 2.71e-11), treg cells (*r* = 0.289, *P* = 4.41e-11), CD56dim cells (*r* = 0.275, *P* = 2.31e-10), and myeloid derived suppressor cells (MDSC, *r* = 0.275, *P* = 2.41e-10). These data indicated that *LIMK1* may play a specific role in immune infiltration in lung adenocarcinoma.

## Discussion

In this study, we found that the mRNA expression of *LIMK1* is upregulated in lung adenocarcinoma tissues. The upregulated mRNA expression of *LIMK1* is positively correlated with lymph node metastases and high TNM stage. ROC curve analysis indicated that *LIMK1* could be a promising diagnostic biomarker to differentiate lung adenocarcinoma from normal tissues. In light of Kaplan-Meier curves and univariate analysis, we confirmed that high mRNA expression of *LIMK1* is associated with short OS and *LIMK1* can be used as a potential biomarker of poor prognosis for lung adenocarcinoma. Moreover, *LIMK1* may play a specific role in immune infiltration in lung adenocarcinoma.

*LIMK1* is one of the members of the LIM kinase family and has been reported to play a significant role in promoting cell invasion and metastasis (Scott and Olson, 2007). Many studies about the oncogenic role of *LIMK1* in several human cancers have been emerged in recent years, including gastric cancer, pancreatic cancer, as well as lung cancer (McConnell et al., 2011; Chen et al., 2013). Furthermore, it is reported that *LIMK1* is upregulated in various cancers and associates with an unfavorable prognosis (Huang et al., 2020). However, the expression of *LIMK1* and its prognostic value has not been fully investigated in lung adenocarcinoma. Here, in this study, based on pan-cancer analysis, our results are consistent with those reports that *LIMK1* mRNA is abnormally expressed in various cancers. We also confirmed that *LIMK1* is significantly upregulated in lung adenocarcinoma. High mRNA expression of *LIMK1* is positively associated with lymph node metastases and high TNM stage, our finding agrees with the previous report by Chen et al. (2013). These findings suggest that *LIMK1* might act as a potential biomarker of poor prognosis to identify lung adenocarcinoma with poor clinical outcome.

Currently, the function of *LIMK1* in tumors had not been fully reported. Previous trials suggest that *LIMK1* may be a target of dasatinib which can inhibit *LIMK1* to suppress lung cancer cell proliferation and growth (Zhang et al., 2020). Other studies have shown *LIMK1* acts as a direct target of miRNA-27-3p and miRNA-128-3p (Chen et al., 2017; Zhao et al., 2019), both miRNA-27-3p and miRNA-128-3p can suppress cancer cell proliferation, migration, and invasion. The underlying mechanism analysis showed that the *LIMK1*-cofilin signaling pathway plays an important role in tumor progression (Nishimura et al., 2006). All these results suggest that *LIMK1* could be regarded as a promising biomarker or emerging target for cancer therapy. Given the condition that mRNA expression of *LIMK1* is significantly higher in lung adenocarcinoma than in normal lung tissues, we speculate *LIMK1* can act as a biomarker to differentiate lung adenocarcinoma from normal controls. In order to validate the clinical value of *LIMK1* in the diagnosis of lung adenocarcinoma, we conducted ROC curve analysis. Our results showed that *LIMK1* had a significantly high AUC value in the detection of lung adenocarcinoma, with 69.5% in sensitivity, 93.2% in specificity, and 71.9% in accuracy. On the basis of our finding, we conclude that *LIMK1* might act as a potential diagnostic biomarker to differentiate lung adenocarcinoma from normal controls.

Recent studies have characterized *LIMK1* as an important biomarker for poor prognosis and associated upregulated mRNA expression of *LIMK1* with poor overall survival in many cancers. In prostate cancer, it is reported that elevated *LIMK1* is positively associated with higher Gleason Scores and incidence of metastasis, as well as poor clinical outcome and reduced survival (Davila et al., 2007; Mardilovich et al., 2015; Huang et al., 2020). In ovarian cancer, Zhang et al. (2012) demonstrated that overexpression of *LIMK1* is significant correlated with severity and poor differentiation level of ovarian cancer. A paper from Zhang et al. (2011) suggested that upregulation of *LIMK1* can promote the invasion and metastasis in drug-resistant osteosarcoma and in turn *LIMK1* can act as a potential novel therapeutic target. In glioblastoma, Chen et al. (2020) reported that *LIMK1* is increased and the overexpression of *LIMK1* is associated with high grade and poor prognosis. In contrast, suppression of *LIMK1* can prolong survival time. However, the prognostic value of *LIMK1* has not been investigated in lung adenocarcinoma. Given the upregulation of *LIMK1* is positively correlated with lymph node metastases and high TNM stage, we speculated *LIMK1* is involved in the development of lung adenocarcinoma. Moreover, since lymph node metastases and high TNM stage are correlated with poor survival, we speculated that the upregulation of *LIMK1* is a biomarker of poor prognosis. Furthermore, in light of Kaplan-Meier curves and log-rank test, lung adenocarcinoma patients with high mRNA expression of *LIMK1* are associated with a decreased survival rate than those with low *LIMK1* levels. On the basis of our data, we concluded that *LIMK1* can be used as a biomarker of poor prognosis for determining prognosis in lung adenocarcinoma.

*LIMK1* is a crucial component of Rac1/PAK1/LIMK1/cofilin signaling pathway, which is involved in several cancers. For example, in cervical cancer, miR-509-3p can regulate this pathway to enhance the apoptosis and chemo-sensitivity of cervical cancer cells (Xu et al., 2012). In gastric cancer, the inhibition of Rho GDP dissociation inhibitor 2 can suppress tumor cell migration and invasion via signaling pathway (Zeng et al., 2020). In this study, co-expression analyses indicated that the expression of *LIMK1* is significantly correlated to that of *Rac1*, *PAK1*, and *CLF1*. On the basis of our finding, we speculate that the upregulation of LIMK1 expression would affect the entire pathway. However, this should be tested in other experiments.

Many studies about the possible role of *LIMK1* in human TILs have emerged in recent years. Xu et al. (2012) reported that *LIMK1* may be involved in spontaneous actin polarization in transformed CD4 T cells. However, the correlation analysis between *LIMK1* expression and immune cell infiltration in lung adenocarcinoma has not been investigated. In this study, we found that several tumor infiltrating immune cells (CD4^+^ T cell, macrophage, neutrophil, dendritic cell) were correlated with the expression of *LIMK1* in lung adenocarcinoma by using TIMER. We also found that positive correlation were indicated between *LIMK1* expression and CD8^+^ T cells, CD4^+^ T cells, monocyte cells, treg cells, CD56dim cells, and myeloid derived suppressor cells. These findings suggest that there is a potential correlation between *LIMK1* and immune infiltration in lung adenocarcinoma. However, further research should be designed to confirm this correlation.

There are several limitations in this study. First, the expression and prognostic implication of *LIMK1* were conducted with online public databases, further study with clinical samples is required to validate these results. Second, to further examine the detailed mechanism of the impact of *LIMK1* on immune infiltration in lung adenocarcinoma, *in vivo*/*vitro* experiments should be designed.

In conclusion, in this study, we showed for the first time that mRNA expression of *LIMK1* is upregulated in lung adenocarcinoma and positively correlated with lymph node metastases and high TNM stage. Our research suggests that *LIMK1* could be regarded as a potential biomarker of poor prognosis to identify lung adenocarcinoma patients with poor clinical outcomes and may play a specific role in immune infiltration.

## Data Availability Statement

The raw data supporting the conclusions of this article will be made available by the authors, without undue reservation.

## Author Contributions

YuZ conceived and designed the study. GL performed data analysis and wrote the manuscript. YiZ and CZ contributed analysis tools. All authors reviewed the manuscript.

## Conflict of Interest

The authors declare that the research was conducted in the absence of any commercial or financial relationships that could be construed as a potential conflict of interest.

## Footnotes

1 https://portal.gdc.cancer.gov/

2 https://proteomics.cancer.gov/programs/cptac

3 http://ualcan.path.uab.edu/

4 https://proteinatlas.org/

5 http://string-db.org

6 https://cistrome.shinyapps.io/timer/

7 http://cis.hku.hk/TISIDB/

8 http://dna00.bio.kyutech.ac.jp/PrognoScan/index.html

9 https://www.r-project.org/

10 https://CRAN.R-project.org/package=survminer

## Supplementary Material

Supplementary Figure 1 The correlation between *LIMK1* and overall survival in two different datasets analyzed with PrognoScan. High expression of *LIMK1* was correlated with poor overall survival in dataset jacob-00182-CANDF **(A)** and jacob-00182-MSK **(B)**.
