# Supplementary figures and images for: Upregulation of LIMK1 Is Correlated With Poor Prognosis and Immune Infiltrates in Lung Adenocarcinoma
Source: Front Genet. 2021 Jun 3;12:671585. doi: 10.3389/fgene.2021.671585 (PMC8209497; doi:10.3389/fgene.2021.671585)

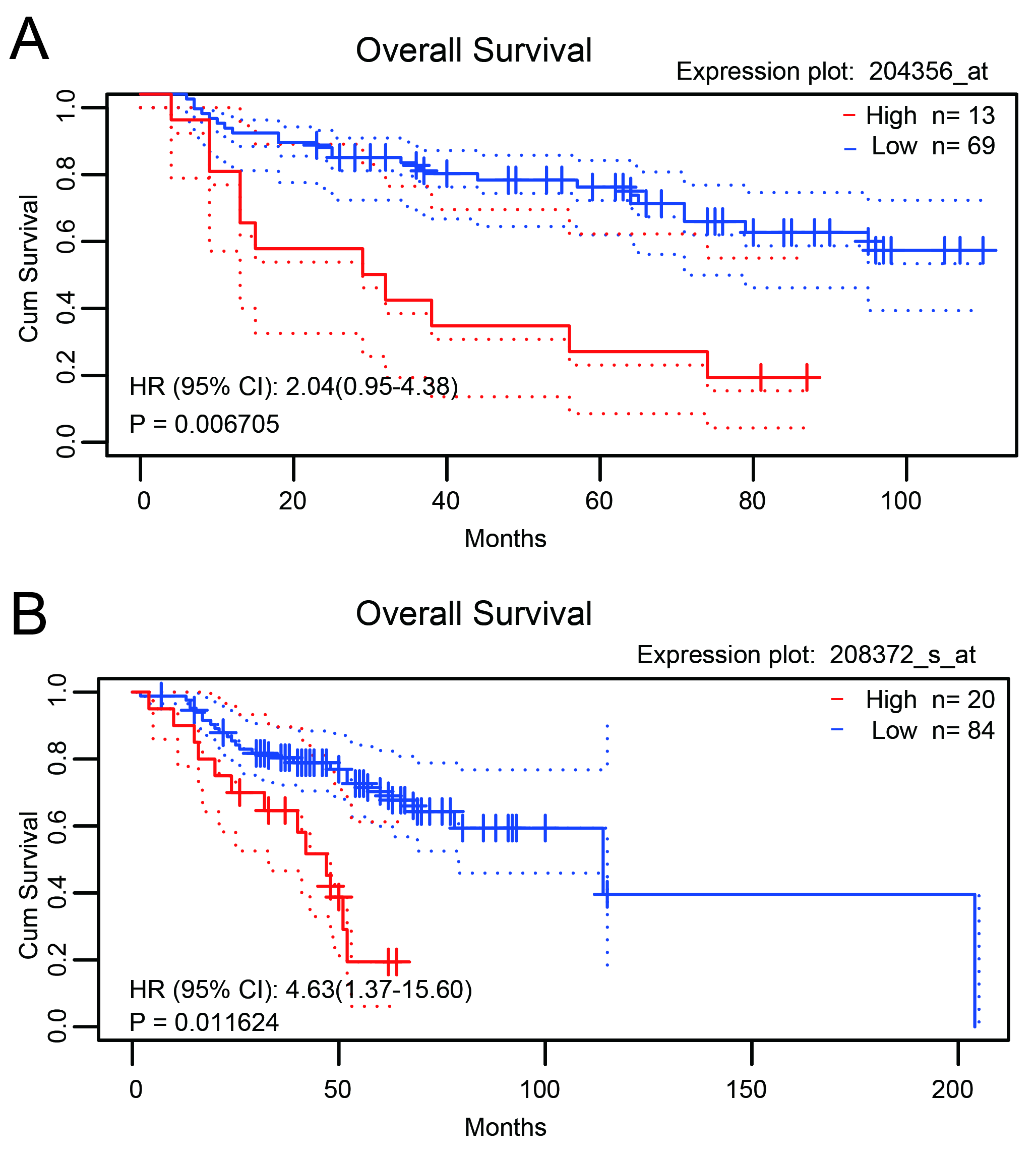

Supplement: Supplementary Figure 1 — The correlation between LIMK1 and overall survival in two different datasets analyzed with PrognoScan. High expression of LIMK1 was correlated with poor overall survival in dataset jacob-00182-CANDF (A) and jacob-00182-MSK (B). [file Image_1.TIF]
