# Supplementary material for: Upregulation of LIMK1 Is Correlated With Poor Prognosis and Immune Infiltrates in Lung Adenocarcinoma
Source: Front Genet. 2021 Jun 3;12:671585. doi: 10.3389/fgene.2021.671585 (PMC8209497; doi:10.3389/fgene.2021.671585)
Supplement: Supplementary file 5 [file Table_4.DOCX]

1. LIMK1 IN UALCAN

<http://ualcan.path.uab.edu/cgi-bin/CPTAC-Result.pl?genenam=LIMK1&ctype=LUAD>

1. TIMER

<http://timer.cistrome.org/>

1. TCGA

<https://portal.gdc.cancer.gov/>

1. HPA

<https://proteinatlas.org/>

1. STRING

<http://string-db.org>

1. TISIDB

<http://cis.hku.hk/TISIDB/>
